# Supplementary material for: Are carbon emissions trading and green financial instruments synergistic? -Comprehensive quantitative research based on content analysis
Source: PLoS One. 2024 Mar 7;19(3):e0298601. doi: 10.1371/journal.pone.0298601 (PMC10919601; doi:10.1371/journal.pone.0298601)
Supplement: S1 Data — (ZIP) [file pone.0298601.s001.zip › Data/地级市Z_F工作报告.zip!/╔╜╢1⁄2/╝├─╧╩╨/└┤╫╘╣1⁄2╓┌║┼í╛┬φ┐╦╩2╛▌═°í┐_2008.docx]

各位代表：

　　我代表市人民政府向大会报告工作，请予审议，并请市政协各位委员和其他列席人员提出意见。

　　一、过去五年政府工作回顾

　　过去的五年，是我市推进省会现代化建设取得显著成效的五年。全市人民以邓小平理论和“三个代表”重要思想为指导，深入落实科学发展观，坚决贯彻国家宏观调控政策，发挥省城优势，发展省会经济，着力改善民生，促进社会和谐，较好地完成了市十三届人大一次会议确定的目标任务。

　　(一)省会经济实力不断增强。国民经济保持平稳较快发展，经济总量和人均水平实现新的提升，2007年，预计完成生产总值2558亿元，人均生产总值5370美元，均比2002年实现翻番。地方财政一般预算收入完成156.7亿元，是2002年的2.37倍。经济结构逐步优化，三次产业比例为5.87：45.07：49.06，现代服务业占服务业增加值比重为43.8%，高新技术产值比重、非公有制经济比重分别达35.3%、39.8%，提高11.3和7.8个百分点。济钢、山水等一批重点企业加快发展，浪潮、力诺等一批高新技术企业茁壮成长，重汽等一批企业摆脱困境焕发生机，成为我市工业经济的支柱。投资结构趋向合理，累计完成固定资产投资4177.5亿元，年均增长23.2%；2007年服务业、高新技术产业、农业和社会事业投资分别是2002年的2.7、2.8、2.3和5.3倍。经济效益明显提高，规模以上工业经济效益综合指数260%，利税、利润分别是2002年的3.6和5.5倍。“十五”计划胜利完成，“十一五”规划进展顺利。全市经济发展朝着又好又快的方向迈进。

　　(二)新农村建设迈出新步伐。认真贯彻中央支持“三农”各项政策，实施支农投入、农民增收“1020”和“6521”工程等系列惠农措施，市财政累计支农投入33.2亿元，是前五年的5.72倍。大力实施新农村建设“十大行动”，农村生产生活条件明显改善，村村通沥青(水泥)路率、通客车率和通自来水率分别达99.1%、99.7%和90.9%；新农合参合率、五保户供养率分别达96%、70%。大力发展现代农业，农业科技推广和服务体系逐步健全，粮食总产268万吨，总产和单产连续五年双增。农业结构调整和产业化经营稳步推进，畜牧、蔬菜、林果产值均有较大提高，规模以上农业龙头企业发展到205家，53%的农户纳入产业化经营。农民人均纯收入6220元，年均增长13.1%，连续四年实现两位数增长。全面取消农业税及附加，农村义务教育各项政策全面落实。实现城乡用电同网同价。农村扶贫取得阶段成效。基础设施延伸、产业转移及中心镇建设步伐加快，章丘等县(市)城市建设发生明显变化，县域财力较快增长，区域发展协调性逐步增强。

　　(三)城乡人民生活进一步改善。城乡居民储蓄存款余额1230亿元，是2002年的1.9倍。城市居民人均可支配收入17794元，年均增长12%，物价保持基本稳定。社会消费品零售总额1100亿元，是2002年的2.5倍。汽车、住房、文化、教育、旅游、健身等支出比重提高。农村居民恩格尔系数下降3.8个百分点。城镇安置就业再就业累计60万人次，登记失业率控制在3.8%以内。转移农村劳动力累计79.4万人次。社会保障门类增加、标准提高、覆盖面扩大，城市居民最低生活保障标准由208元提高到280元，农村居民最低生活保障水平有新的提高，城乡居民享受最低生活保障人数分别达78.4万人次和37万人次。住房保障体系逐步完善，住房公积金制度覆盖面扩大，大力推动棚户区改造，经济适用房、拆迁安置房累计竣工330万平方米，廉租房制度受助家庭累计8000户次。农民居住条件得到改善，城市居民人均住房使用面积21平方米，比2002年增加3.2平方米。

　　(四)经济发展方式加快转变。大力推进自主创新，着力抓好节能减排，创新型城市建设、资源节约型和环境友好型社会建设迈出实质步伐。鼓励自主创新的体制政策日趋完善，“政企产学研金”自主创新模式逐步形成，知识产权保护力度加大。累计研发投入167.3亿元，取得重要科技成果1226项，申请专利30420项，新增省级以上企业技术中心29个、工程技术研究中心48个、驰名商标12个。跻身中国服务外包基地城市、国家软件出口创新基地和国家知识产权工作示范城市行列，国家信息通信国际创新园落户济南。严格限制“两高”产业发展，加快淘汰落后产能，实施百项重大节能示范工程，涌现出一批循环经济亮点，万元生产总值能耗、主要污染物排放强度均呈下降趋势。生态环境不断改善，2007年空气质量良好以上天数比率达85%以上。地下水水质良好，河道截污和污水处理工程加快推进。开发复垦整理土地29.2万亩，恢复治理矿山572万平方米，推进南部山区水源涵养和生态保护建设，全市森林覆盖率达26.6%，提高6.3个百分点。

　　(五)城市面貌发生明显变化。实施“东拓、西进、南控、北跨、中疏”城市空间战略和“新区开发、老城提升、两翼展开、整体推进”发展思路，城市发展框架基本拉开。完成新一轮城市规划修编，编制完成南部山区分区规划和重点区域控制性规划、北跨地区发展战略规划，中心城区首次实现控制性详细规划全覆盖，新农村建设规划全面展开，城乡规划体系基本形成。累计投资1179.2亿元，实施城市建设“十大工程”，加快推进城市基础设施建设，经十路、经一路、济泺路、旅游路、顺河高架路北延、济菏和济莱高速、济南机场扩建等一批重点工程相继建成，高标准完成104国道、102和103省道路段改造。高新区新区、大学科技园初具规模。奥体中心、绕城高速大北环、济阳黄河大桥、北园大街快速路等重点工程加快推进。大明湖扩建、小清河综合整治工程相继开工。改善背街小巷面貌环境，对违法违章建设、破损山体和市容环境展开全面整治。供电能力提高76%，连续10年零拉路限电。城市集中供热普及率、燃气气化率、绿化覆盖率分别达52.6%、98.5%和36.8%，新增绿化面积1652万平方米，人均公共绿地9.5平方米，提高21.8%。节水保泉成效显著。推动城市管理重心下移，初步形成“两级政府、三级管理、四级网络”城市管理体制，构筑起城市规划和重点工程建设制度体系。积极推进市政环卫作业市场化，全面推行城市管理综合执法，网格化数字化管理平台初步形成。

　　(六)各项社会事业全面发展。不断强化社会管理和公共服务，累计完成社会事业投资472亿元。努力促进教育公平，教育管理体制改革不断深化，教育布局结构趋向合理，农村教育振兴行动成效显著。农村义务教育“两免一补”全面落实，城市义务教育阶段学杂费全部免除，困难家庭学生免除课本费、补助寄宿生活费，建立起高等教育、中等职业教育和高中阶段家庭困难学生资助政策体系，在全国率先取消外来务工子女借读费，进城务工子女教育问题得到有效解决。适龄儿童入学率、小学毕业生升学率、小学在校生巩固率均达100%，高中阶段教育普及率提高到90.1%，职业教育和成人教育蓬勃发展，普通高等教育在校生44万人，是2002年的2.1倍。抗击非典取得胜利，公共卫生体系不断健全，市疾病预防控制中心建成使用，县(市)区全部建立疾病预防控制和医疗救治体系，全面完成乡镇卫生院改造，新建社区卫生服务机构36个。在全省率先实施“惠民医疗工程”。文化事业和文化产业加快发展，推出一批文化艺术精品，荣获国际金奖6项、省以上奖励800余项。济南国际幽默艺术周、泉城大舞台等成为知名文化品牌。物质文化遗产、非物质文化遗产保护以及考古发掘取得新成果。全民健身蓬勃开展，竞技体育水平不断提高，获省级以上比赛金牌915.5枚，成功承办亚足杯等大型赛事，第十一届全运会主赛区筹备工作顺利推进。大力实施人才强市战略，人才队伍建设成效显著，科技人才和企业家队伍不断壮大。人口自然增长率控制在5‰以内，人口素质进一步提高。新闻出版、广播电视、社会科学、外事侨务、民族宗教、档案史志、妇女儿童、社会福利、老龄、残疾人、慈善事业、气象、防震减灾等工作取得新成绩。

　　(七)改革开放取得新进展。着力深化重点领域和关键环节改革。围绕转变政府职能，实施行政机构和行政审批制度改革，精简职能错位交叉机构31个，依法取消行政许可事项695项，对政府规章、规范性文件、执法行为和收费项目作了全面清理；实施政府资金、行政事业资产管理制度改革，规范行政支出，降低行政成本；实施政府投融资体制改革，提高资金使用效益，防控财政风险；实施财税体制和统计制度改革，激发区域发展活力。完成乡镇机构改革，农村综合改革扎实推进。完善国有资产监管体制，加大国企改革重组力度，上市公司实现股权分置，支持重汽在港成功上市，完成粮食流通体制改革，推动公用事业改革步伐，民营经济健康发展。开放型经济步伐加快，利用外资规模和质量提高，累计实际利用外商直接投资15.3亿美元，年均增长19.4%，现代服务业、基础设施实际利用外资增长较快。对外贸易迈上新台阶，2007年实现进出口总值60.5亿美元，出口33.5亿美元，年均分别增长32.2%和37%，机电产品、高新技术产品出口比重加大。“走出去”取得积极进展，科教文卫等领域对外交流不断扩大。

　　(八)和谐济南建设扎实推进。坚持依法行政，推进法治政府建设，行政执法责任制全面落实，民主科学决策水平进一步提高。及时向人大常委会报告工作，更加注重同人民政协的联系，自觉接受人大和政协监督，累计办理人大代表建议1645件、政协提案2525件。社会管理和基层服务网络逐步健全，社区居委会和村委会建设得到加强，涌现出青年公园街道应急机制进社区、泉城义工等便民服务典型。“五五”普法全面启动，法律服务体系日益健全，公民法律意识显著增强。群众性文明创建活动广泛开展，公民思想道德建设加强，“诚信、创新、和谐”的城市精神深入人心，创城“百件实事”成效显著。高度重视信访工作，加强矛盾纠纷排查调处，积极预防、疏导和化解社会矛盾。市场和产品质量监管得到加强，食品药品监管工作取得新的成效。深入推进平安建设，依法打击各类犯罪活动，社会治安秩序总体良好。经受“7·18”暴雨洪灾重大考验，处置应急突发事件能力逐步增强。深入开展安全生产专项整治活动，安全生产形势基本稳定。人民防空建设不断加强，国防教育和双拥共建成效显著，荣获全国双拥模范城“五连冠”。注重从源头上预防和治理腐败，从体制、机制和教育上强化措施，廉政建设和反腐败斗争不断深入，加强监察审计监督和制度建设，在查处腐败案件、治理商业贿赂、纠正损害群众利益不正之风等方面取得新进展。

　　各位代表！五年的发展变化，凝聚着全市人民的智慧和汗水。五年中，我们遇到的困难比预料的大，取得的成绩比预想的好。面对经济体制转轨和社会转型加快、矛盾增多的复杂局面，非典、禽流感、暴雨洪灾等重大考验，全市上下同心同德、奋力拼搏，突破一个又一个难点，推动省会现代化建设不断前进。五年来的发展和进步，是省委、省政府和市委正确领导的结果，是全市人民艰苦奋斗、开拓进取的结果，是省城各界团结一致、共同努力的结果。在此，我代表市政府，向辛勤工作在各行各业的广大干部群众，向给予政府工作大力支持的各级人大代表、政协委员、各民主党派、工商联、各界人士，向帮助支持省城建设的人民解放军驻济部队、武警部队官兵，中央、省驻济机关单位，向为济南发展作出贡献的港澳台同胞、海外侨胞、国内外朋友，表示崇高的敬意和诚挚的感谢！

　　过去的五年，我们取得了很大的成绩，但是也应清醒地认识到，我市在经济社会发展中还存在一些突出问题和矛盾。主要是：思想观念和工作方式还不能很好地适应科学发展的要求，推进科学发展的能力需要进一步增强；转变经济发展方式的步伐还不够快，经济结构不尽合理，节能减排形势严峻；社会事业发展相对滞后，公共服务有待改善，城市安全运行有待加强；群众生活中的一些切身利益问题还没有解决好，城乡部分群众生活还比较困难；政府工作人员服务意识、办事效率仍有差距，发展环境亟待进一步改善;极少数领导干部腐败问题影响很坏，教训十分深刻。对此，我们必须高度重视，努力从根本上加以解决。

　　二、今后五年工作的基本思路和主要目标

　　今后五年是我市全面建设小康社会的关键时期，也是推进省会现代化建设必须紧紧抓住、大有作为的重要机遇期。党的十七大明确提出全面建设小康社会的新要求，省、市九次党代会确立了未来五年经济社会发展的宏伟蓝图，“9·29”省委常委扩大会议提出今后两年城市发展的奋斗目标。这对政府工作提出了更高的要求和期望。我们要全面认识工业化、信息化、城镇化、市场化、国际化深入发展的新形势新任务，进一步增强落实科学发展观的坚定性和自觉性，凝神聚力，艰苦奋斗，更好地肩负起加快省会现代化建设的重要使命。

今后五年政府工作的基本思路是：全面贯彻党的十七大精神，高举中国特色社会主义伟大旗帜，以邓小平理论、“三个代表”重要思想为指导，深入贯彻落实科学发展观，加快工作指导转变，切实履行“维护省城稳定，发展省会经济，建设美丽泉城”神圣职责，致力于改善和保障民生，全面加强经济、政治、文化、社会建设，努力推进科学发展、和谐发展、率先发展，为提前实现全面建设小康社会的目标而努力奋斗。

　　今后五年经济社会发展的主要预期目标是：

　　——— 综合实力再上新台阶。在优化结构、提高效益、降低消耗、保护环境基础上，生产总值突破4500亿元，人均生产总值比2000年翻两番。地方财政一般预算收入年均增长15%。县域经济实力明显增强，城乡区域发展更趋协调，省会城市群经济圈建设迈出实质步伐，在全省“一体两翼”发展格局中发挥更大作用。

　　——— 经济发展方式明显转变。自主创新能力显著提升，科技进步对经济增长的贡献率达55%以上。经济结构明显优化，构筑起以服务经济为主的产业结构。生态文明建设迈出坚实步伐，单位生产总值能耗降到1吨标准煤以下，主要污染物排放在完成“十一五”目标基础上进一步下降，初步形成节约能源资源和保护生态环境的产业结构、增长方式和消费模式。

　　——— 人民生活迈向全面小康。人民富裕程度普遍提高，城市居民人均可支配收入和农村居民人均纯收入年均增长8%以上，物价总水平保持基本稳定。社会就业更加充分，收入分配更加公平，教育公益性更加突显，公共卫生服务体系更加健全，人人享有基本生活保障。逐步实现学有所教、劳有所得、病有所医、老有所养、住有所居的目标。

　　——— 改革开放取得新的突破。市场经济体制不断完善，现代市场体系逐步健全，国有经济活力和竞争力显著增强，非公有制经济加快发展。行政管理体制改革继续深化，法治政府制度框架基本形成，公共服务和经济调节能力不断提高。发展环境明显优化，开放型经济、对外交流合作、城市国际影响力显著提升。

　　——— 城市发展更富特色魅力。努力打造“泉水之都”城市品牌，生态环境明显改善，市区主要河道水质达标，空气质量良好率继续提高。新区建设与老城提升全面加快，城市载体功能显著增强，城市管理更富人性化，初步构筑起泉城特色鲜明、现代气息浓郁的省会城市框架。

　　——— 和谐社会建设更加深入。社会管理体制更加完善，基本公共服务更趋均等。城市文明程度不断提高，基层民主进一步扩大，全社会法制观念增强，公共安全得到有效保障，人民群众拥有更多知情权、参与权、表达权、监督权，形成和睦团结、共建共享和谐社会的生动局面。

　　经过全市人民不懈努力，省会现代化建设迈出更加坚实的一步，经济发展更有活力，生态环境更加良好，城市功能更加健全，文化发展更加繁荣，社会发展更加和谐，人民生活更加殷实，省城人民期盼的繁荣、文明、和谐、宜居的新泉城，必将展现在我们面前！

　　各位代表，实现上述目标，任务光荣而艰巨。在工作指导和把握上努力做到四个坚持：第一，坚持以科学发展观统领全局，用中国特色社会主义理论武装头脑、指导实践、推动工作，把发展作为第一要务，把握发展规律，转变发展方式，增强发展的协调性，努力促进又好又快发展。第二，坚持以人为本执政理念，始终把实现好、维护好、发展好最广大人民的根本利益作为一切工作的出发点和落脚点，着力解决民生突出问题，尊重群众主体地位，发挥群众首创精神，保障群众合法权益，促进人的全面发展。第三，坚持改革开放不动摇，积极推进重点领域和关键环节改革，切实把握好改革发展稳定的关系，使各项改革决策更加符合民心民意，在更高层次上推进对外开放，努力构筑充满活力、富有效率、更加开放、有利于科学发展的体制机制。第四，坚持求真务实作风，不断解放思想，提升境界，推进管理创新，勇于破除一切不符合科学发展观要求的思想观念和工作方式，脚踏实地把各项事业不断推向前进。

　　三、全面做好2008年各项工作

　　2008年是全面贯彻落实党的十七大精神和省市九次党代会工作部署的第一年，也是改革开放30周年，又是新一届政府履行职责的开局之年。做好今年的工作，对于加快实现全面建设小康社会奋斗目标具有重要意义。

　　今年经济社会发展主要预期目标是：(1)经济保持平稳较快增长。生产总值增长12%。地方财政一般预算收入增长16%。固定资产投资增长20%。社会消费品零售额增长15%。外贸进出口增长15%，实际利用外商直接投资增长15%。(2)经济结构进一步优化。服务业增加值所占比重提高1个百分点以上，现代服务业占服务业增加值比重提高到45%左右。高新技术产值比重提高3个百分点。(3)节能环保取得新进展。单位生产总值能耗降低4.6%，二氧化硫、化学需氧量排放分别削减2.38%和3.62%。(4)社会事业全面发展。城镇安置就业再就业13万人次，转移农村劳动力16万人。城镇登记失业率控制在3.8%以内，人口自然增长率控制在5‰以内。(5)人民生活进一步改善。城市居民人均可支配收入和农民人均纯收入增长8%。居民消费价格总水平涨幅控制在上年全市平均水平之内。

　　做好今年各项工作，必须更加坚定地落实国家宏观调控政策，坚持稳中求进、好字优先，贯彻中央“两个防止”的要求，适应稳健的财政政策和从紧的货币政策，着力加强影响科学发展和改善民生的薄弱环节，努力实现速度质量效益相协调、消费投资出口相协调、人口资源环境相协调、改革发展稳定相协调，保持经济社会又好又快发展势头。

　　(一)加快推进经济发展方式转变

　　大力提高自主创新能力。坚持把自主创新作为城市发展战略的核心，加快推动经济发展由资源依赖型向创新驱动型转变。全面落实中长期科学和技术发展规划纲要，发挥政策激励导向作用，引导鼓励全社会增加自主创新投入。举全市之力加快高新区发展，充分发挥国家信息通信国际创新园等品牌作用，整合省会创新资源，加强创新平台建设。以国民经济、社会发展和人民生活急需科技支撑的领域和环节为重点，实施一批带动力强的重大科技专项，力求在电子信息、新能源、新材料等领域实现突破。完善“政企产学研金”发展模式，培育以企业为主体的自主创新体系，激发企业创新活力。实施知识产权战略，引进培养创新型适用人才，完善风险投资等金融服务体系，为自主创新提供有力支撑。

　　积极推动经济结构优化升级。以发展高新技术产业、先进制造业和现代服务业为重点，深入推进新型工业化，加快构筑以服务经济为主的产业体系。把繁荣发展服务业放在更加突出的位置。通过规划引导、政策推动、开放带动多措并举，着力提升金融、物流、信息、会展、中介等生产性服务业，突出发展软件、文化、教育培训等知识型服务业，大力发展旅游、商贸、社区服务等生活性服务业。发展总部经济、创意经济，提升城区服务业发展水平。加快建设金融商务核心区和现代物流园，提升齐鲁软件园等服务业重点区片。规范和引导房地产业健康发展。大力发展高新技术产业和先进制造业。推进信息化与工业化融合，实施创新、品牌、开放、园区带动，以交通装备、电子信息、石油化工、冶金钢铁、机械装备、食品药品等产业为基础，突出发展以集成电路、软件和新型元器件为重点的信息产业，以太阳能光伏光热为重点的新能源产业，以有机高分子材料、高性能金属材料、电子发光材料、环保节能建筑材料为重点的新材料产业，以清洁能源轿车和重型汽车为重点的交通装备产业，以光机电一体化产品、柔性加工中心为重点的机械装备产业，以汽轮机、变压器为重点的电力装备产业，以中药、生物制药为重点的医药产业，提高研发能力，培植龙头企业，延伸产业链条，大力提高工业规模实力、质量效益、产业集中度和创新能力。

　　加强能源资源节约和生态环境保护。把节能减排作为转变发展方式的切入点和重要标志，大力推进生态文明建设。一是优化结构推进节能减排。切实把完成节能减排任务建立在产业结构调整基础上，加快构建节能环保型产业体系。严控新上“两高”项目，加快关停小火电机组进度，淘汰机立窑水泥熟料产能45万吨，实施裕兴化工厂等城区工业企业搬迁改造。发展太阳能、生物质能、地热等新能源，大力发展循环经济，鼓励发展再生资源产业。二是突出重点推进节能减排。抓好100户重点企业节能减排，深入实施10大重点节能工程，提高工业、交通、建筑、服务业等重点行业节能水平，实施河道截污、垃圾污水处理、烟气脱硫等一批重点工程，突破和推广一批重大节能技术，加快工业采用地表水和中水回用步伐，推进县和重点镇污水处理设施建设。三是以管理创新推进节能减排。发挥政策导向作用，引导企业和社会加大节能环保投入。重视运用价格、财税手段推进节能环保，依法查处严重浪费资源和危害环境的行为。健全节能减排统计、监测、考核体系，严格落实目标责任，对没有完成节能减排任务的实行一票否决！

　　坚决落实国家宏观调控政策。把握控总量、稳物价、调结构、促平衡的基调，落实“两个防止”的措施。努力扩大消费需求，大力优化投资结构。严把新上项目关口，引导鼓励社会资金重点投向服务业、高新技术产业、先进制造业、现代农业、社会事业和节能环保产业。严格土地调控和监管，盘活存量，提高增量土地利用效率，整合可利用土地资源，更好地支撑和服务经济发展。采取有力措施，切实保障市场供应和维护市场经济秩序，优化财政支出结构，继续增加对社会保障、卫生、教育、住房保障等方面支出，自觉维护好宏观调控大局。

　　(二)进一步保障和改善民生

　　从解决群众最关注的问题入手，切实履行政府公共服务职责。一是优先发展教育。坚持教育公益性质，加大教育投入，促进教育公平，推动各类教育协调发展，提高素质教育水平，努力让更多的人接受更好的教育。健全学生资助制度，解决好经济困难家庭、进城务工人员子女就学难问题。二是进一步扩大社会就业。健全覆盖城乡的职业技术培训制度，完善市场就业促进机制，以创业带动就业，扩大就业规模，改善就业结构，全面提高劳动者就业技能。以贯彻《劳动合同法》、《就业促进法》为契机，规范改善劳动关系，提高就业稳定性，切实保障劳动者合法权益。完善困难群体就业援助制度，重视解决退伍军人、大学生、新生代农民工就业问题。着力提高低收入者收入，扩大中等收入群体，逐步缩小收入分配差距。三是提高社会保障水平。以社会保险、社会救助、社会福利为基础，以基本养老、基本医疗、最低生活保障制度为重点，以慈善事业、商业保险为补充，加快建立覆盖城乡居民的社会保障体系。切实管好用好社保基金，逐步做实养老保险个人账户。继续增加财政保障性投入，全市企业职工基本养老、医疗、失业、工伤、生育保险参保人数分别达103万、105万、70万、100万和61万，农村养老保险参保45万人。积极发展慈善事业，完善社会救助机制，加快老年服务、重度残疾人服务等各类福利设施建设，确保城乡弱势群体基本生活。四是全面加强城乡医疗卫生服务。深化医疗卫生体制改革，完善经费保障机制，推进农村卫生室建设和社区卫生服务设施改造，搞好农村和社区医疗人员培训，实施城市医疗人才支农行动，提高基层医疗服务水平。启动城镇居民基本医疗保险试点工作，提高新农合筹资水平。健全疾病预防控制体系，提高应对突发公共卫生事件应急处理能力。五是继续做好住房保障工作。进一步推行住房公积金制度，健全以廉租住房制度为重点、增加经济适用房供给、多渠道解决城市低收入家庭住房困难的保障体系。完成今年棚户区改造任务，加快推进“城中村”整合改造，多途径加强住房保障建设，全面改善城乡居民居住条件。新区建设、老城改造都要重视居民生活必需设施的配套建设。对中央和省出台的各项民生政策，要不折不扣贯彻落实，结合实际今年集中力量办好12件实事。特别要适应新的情况，加强市场监管，稳定主要食品供应和市场价格，通过增加低收入群体收入、提高低保标准、建立消费价格指数与价格补贴联动机制等措施，确保低收入家庭生活水平不因价格上涨而降低。

　　(三)推进城乡区域协调发展

　　始终把解决“三农”问题放在全面建设小康社会突出位置，健全以工促农、以城带乡长效机制，推动城乡一体化发展。一是大力发展现代农业。积极推进精致高效农业、休闲观光农业、设施农业发展，加快构筑生产、生活、生态融合的都市型农业发展体系。着力构筑产业、经营、科技、标准、投入五大支撑体系，落实粮食安全“三条底线”，大力推进农业结构调整，继续做强畜牧业、奶业，做优蔬菜产业，做大林果苗木花卉产业，培育章丘大葱、商河大蒜、平阴玫瑰、历城核桃等特色农产品基地。规模以上农业龙头企业达到210家，农业科技进步贡献率达到53%以上，54%的农户纳入产业化经营。积极推进标准化生产，加强动植物疫病防控，提高农产品质量安全水平。二是促进农民持续增收。完善农民增收长效机制，继续开展农民培训“五大工程”，通过农业内部挖潜、发展非农产业、促进劳动力转移等途径，拓宽农民增收渠道。三是深入推进新农村建设。严格落实“三个高于”支农惠农政策，市财政支农投入14亿元，加快实施“十大行动”，着力完善统筹城乡的基础设施、公共服务设施和生活服务设施，加快农田水利设施建设和黄河标准化堤防建设，实施“百库除险”和河道治理工程，发展改善节水灌溉10万亩，搞好80平方公里小流域水土保持综合治理，推进小城镇起步区建设。四是完善农村市场和服务体系。大力发展农民专业合作组织，探索建立企业、合作社与农户利益联结机制，推进农村市场流通和金融服务体系建设，鼓励各类人才到农村创业发展。

　　把县域经济摆在统筹城乡区域发展的重要位置。一是推进县区经济加快发展，统筹城乡资源，优化产业布局，完善体制政策，加强分类指导，鼓励近郊区做大做强，支持远郊县迎头赶上。突出抓好道路、桥梁、供水、供电、节能减排等重大基础设施向农村延伸。完善促进要素流动的体制政策，中心城区重点发展现代服务业和高新技术产业，推动传统产业向郊县转移。发挥重点工业园区带动作用，明确产业定位，大力提高承载力和招商引资水平。二是启动统筹城乡发展综合改革试点。编制规划、制定政策、健全机制，着力突破统筹城乡发展的体制政策难点。推动基础设施建设和社会事业发展，搞好小城镇建设，促进产业和生产要素合理布局，统筹城乡就业和社会保障。稳定农村土地经营制度，有条件的地方发展多种形式适度规模经营。三是实施重点帮扶措施。加大对财政困难县区转移支付力度，实施重点帮扶，推动县域经济协调发展。提高扶贫开发水平，努力缩小城乡区域发展差距。

　　(四)以迎全运会为契机提升城市功能面貌

　　举办第十一届全运会，是我市城市发展的重大机遇。必须紧紧把握这一契机，全面带动城市规划建设管理，努力使市政建设和市容市貌有一个大提高、上一个大台阶。一是大力提高城市承载力。切实增强规划的权威性、严肃性，发挥好规划的引导调控作用。集中打造泉城特色标志区、奥体文博等重点片区，增强功能，彰显特色，提升形象。加快推动大学科技园、西客站、小清河片区建设，带动城市西部和北部发展。加快构筑布局合理、功能完善、便捷高效的基础设施体系，保障城市安全运行。高质量完成奥体中心、全民健身中心等一批体育设施建设。实施城市综合交通规划，完善城市路网骨架，优先发展公共交通，建成北园大街快速路，启动二环东路等快速路和公交场站枢纽建设。按照“一环八射”高速路网和“十横九纵八连”干线公路网规划，尽快启动济南—商河高速路建设，改造220国道长清平阴段、104国道济阳段。加快建设济阳黄河大桥、黄河三桥、建邦大桥等“北跨”交通设施。推进京沪高铁济南段和西客站枢纽建设。支持航空事业加快发展，争取开辟新的国际国内航线。集中力量搞好大明湖扩建和小清河综合整治工程。实施电网扩能建设改造、黄台电厂以大代小供热、禹城—崔寨天然气专线、清源湖水库和东联供水等一批工程，提高城市“四供”能力。加快腊山分洪等城市防洪设施建设。开工建设第三污水处理厂。二是全力搞好城市综合整治。抓住严重影响城市形象的突出问题，点、线、片结合，深入推进“八乱”整治。完成重点区域破损山体治理，全面整治违法违章建设。对城区重点路段、广场、公园和桥梁实施灯亮工程，实现城市街巷路灯亮化全覆盖。对大辛河、兴济河、工商河等市区主要河道实施截污治污，加强汽车尾气、扬尘治理和渣土管理，采取综合措施实现农作物秸秆全面禁烧。规划建设改造一批停车和市场设施，疏堵结合解决占道经营问题。三是提升城市管理水平。强化区级属地管理责任，完善责权统一的运行机制，切实把工作重心转到社区服务和城市管理上来，推动城市管理由粗放型、突击式向精细化、常态化、数字化、法制化转变。实施小区环境综合整治，规范加强物业管理。提升交通管理水平，缓解交通阻塞问题。完善城郊村居改造政策，依法推进村居建设。四是加强生态资源保护。落实南部山区规划，完善政策措施，统筹推进南部发展，坚决遏制乱开发乱建设。实施城乡造林绿化工程，加快黄河沿线绿色风貌带建设，森林覆盖率提高1个百分点，实施南部山区人工降雨工程建设，加强水资源管理和水源涵养。强化节水保泉长效措施，确保正常年份泉水持续喷涌，推进泉水先观后用，努力建设天蓝水清的生态环境。

　　(五)扎实推进文化强市建设

　　以保障人民群众文化权益为目标，大力推动文化建设。一是繁荣发展文化事业。制定实施文化发展专项规划和文化强市建设实施纲要，发展哲学社会科学，繁荣文艺创作和群众文化，努力打造体现地域文化特色和时代精神的文艺精品，推出一批有影响力的优秀剧目，办好第五届中国京剧节。努力建设结构合理、覆盖城乡、惠及全民的公共文化服务体系。通过政府采购方式，开展公共文化服务活动，活跃社区和农村文化生活。二是积极发展文化产业。加快文化产业基地和区域性特色文化产业群建设，广泛运用信息技术，促进广播影视、新闻出版、文化旅游、文化创意、文化博览等产业发展，培育一批有实力的文化、传媒集团，开发健康文明的网络文化、音像、演出、娱乐、健身、艺术品、动漫等产品，积极开拓演出市场。以“泉水之都、济南旅游”为主题，整合自然历史文化资源，构建大旅游产业，大力发展商务会展、节庆旅游，培育区域旅游新优势。实施泉水文化品牌带动和文化“走出去”战略，提升我市文化竞争力。三是深化文化体制改革。完善公益性文化事业、文化产业、文化创新扶持政策，营造有利于出精品、出人才、出效益的环境。继续深化文化事业内部改革，增强文化艺术团体发展活力。加快文化产业结构调整，建立多元投入机制，鼓励非公有资本进入文化产业，推动文化艺术资源整合提升。四是突出抓好公共文化设施建设。以基层图书馆、博物馆、文化馆为重点，构建配套完善的市、县、乡、村四级公共文化设施网络。面向社区和农村，着力抓好广播电视村村通、综合文化站建设、文化信息资源共享、农村电影放映、农家书屋“五大工程”。五是加强历史文化名城、历史文化街区、文保单位保护。规划建设一批体现省会和泉城特色的标志性文化设施，推进府学文庙、城子崖遗址、洛庄汉王陵博物馆建设。抓好非物质文化遗产保护、挖掘、整理和传承，完成济南通史编纂，道路命名、城市设计、建筑风格更多体现地域文化特色。六是深入开展“迎和谐全运、建美丽泉城”主题行动。全面做好全运会筹备各项服务工作，大力开展全民健身活动，推动体育产业发展。加大社会动员力度，开展志愿者行动，落实接待服务、综合保障、治安保卫各项措施，努力创造一流的设施服务环境，大力加强城市窗口和市民文明素质建设，形成全社会关心支持全运会的浓厚氛围。

　　(六)进一步深化改革扩大开放

　　深化国有企业公司制股份制改革，做大做强骨干优势企业。完善国有资本出资人代表派出机制和经营预算体系，提高国有资本运营效率，推进国有资源和行政事业国有资产有偿使用改革。认真落实非公经济发展各项政策，破除体制障碍，推进公平准入，改善融资条件，推进民营经济加快发展。完善现代市场体系建设，大力发展土地、资本、技术、人才、劳动力和产权交易等要素市场，构建反映市场供求关系、资源稀缺程度、环境损害成本的生产要素和资源价格形成机制。以岗位设置管理和人员聘用制度改革为重点，加快事业单位改革。规范发展行业协会和中介服务机构，深化社会信用体系建设。

　　全面提高开放型经济水平。转变外贸增长方式，调整出口结构，大力发展服务贸易，重点发展服务外包，促进加工贸易转型升级，鼓励支持低能耗、环保型产品和有自主品牌的高新技术产品、机电产品出口。改进招商方式，完善项目推进机制，优化投资环境，切实提高利用外资质量和水平。以高端产业为重点，进一步加大利用外资力度，引导外资更多投向基础设施、高新技术产业、先进制造业和现代服务业领域，发挥外资在推动自主创新、产业升级、区域协调发展等方面的积极作用。鼓励支持有条件的企业境外投资设厂、上市融资，开展国际化经营。坚持“引资”、“引智”并举，吸引海外人才来济创业。培育技术型劳务资源，改善外派劳务人员结构。拓宽文化、科技、体育、旅游、城市管理等领域对外交流合作。加强口岸建设。更多举办对外宣传和涉外活动，扩大国际影响力。

　　(七)积极促进社会和谐稳定

　　推进社会主义民主法制建设，健全民主制度，发展基层民主，加强城乡基层自治组织建设，推进厂务公开、村务公开，切实维护群众民主权利。加快推进依法治市，加强法制宣传教育，形成学法、用法、守法良好社会氛围。深入推进平安济南建设，加强社会治安综合治理，健全社会治安防控体系，依法打击各类犯罪活动，严厉查处非法集资、传销、诈骗等破坏市场经济秩序的案件，维护省城安定团结，不断增强人民群众的安全感。高度重视信访工作，认真解决群众合理诉求，依法妥善处理各类社会矛盾和利益冲突。加强公共安全和应急管理工作，完善突发事件应急管理体制。加强人防设施建设，实现人防到民防的转变。组织济南解放60周年纪念活动，加强国防教育，深入开展双拥共建活动，增进军政军民团结，继续争创双拥模范城。坚持计划生育基本国策，稳定低生育水平，提高出生人口素质，积极应对人口老龄化，切实加强流动人口服务和管理。高度重视安全生产，突出抓好矿山企业、危险化学品、交通、建筑工地和人员聚集场所安全管理，坚决防止重特大事故发生。继续整顿和规范食品药品市场秩序，加强餐饮卫生监管，让人民群众吃上放心食品，用上放心药品。深入抓好全国文明城市创建工作，继续实施创城主题行动。加强社会主义核心价值体系建设，大力实施公民道德建设工程。加强网络管理，开展心理健康教育，积极营造健康文明的网上舆论氛围。

　　四、建设人民满意的公共服务型政府

　　按照社会主义政治文明建设要求，以建设公共服务型政府为方向，用改革创新的精神，加强政府建设，推进管理创新，更好肩负起省会现代化建设的历史责任。

　　(一)强化公共服务职能，完善经济调节机制。

　　适应完善公共财政体系的新要求，加强财税工作和财源建设，实现财税收入稳定增长。深化预算管理制度改革，调整财政支出结构，优化公共资源配置，完善公共服务体系，加大改善民生的投入，推进基本公共服务均等化。以转变职能、理顺关系、优化结构、提高效能为目标，按照权责一致、分工合理、决策科学、执行顺畅、监督有力的方向，继续深化行政管理体制各项改革，不断巩固扩大改革成果，降低行政成本，提高行政效能。以和谐社区建设为切入点，加强社会建设，支持社会组织和群众参与社会管理，形成政府主导、社会协同、公众参与、共建共享的社会管理格局。深入推进政企、政事分开以及政府与市场中介组织分开，精简和规范行政审批。健全经济调节职能，注重规划和政策引导，完善导向激励约束机制，运用经济、法律和必要的行政手段，确保国家宏观调控政策落到实处。以完善设施、健全法制、加强服务、提高效能为重点，大力优化发展环境。

　　(二)坚持民主科学决策，深入推进依法行政。

　　健全民主科学决策制度，完善重大问题集体研究制度和决策前民主协商制度、专家咨询制度、公众意见征集、社会公示和听证制度。贯彻国务院《政府信息公开条例》，继续深化政务公开，完善办事公开制度，搞好电子政务建设，提高政府工作透明度和公信力。加强政府法制工作，重点推进社会事业、社会保障、社会管理、节约环保等方面规章建设。加强和改善行政执法，严格按照法定权限和程序行使权力、履行职责。进一步加强行政监督，自觉接受人大及其常委会监督、政协民主监督，认真听取民主党派、工商联、无党派人士和各人民团体意见，接受新闻舆论和社会公众监督。做好行政复议、法律服务和法律援助工作。按照《公务员法》要求，加强政府系统公务员考核、监督和管理。

　　(三)规范行政权力运行，加强反腐倡廉建设。

　　把反腐倡廉放在政府建设更加突出的位置，坚持标本兼治、综合治理、惩防并举、注重预防的方针，推进惩治和预防腐败体系建设。在坚决惩治腐败的同时，更加注重治本，更加注重预防，更加注重制度建设，拓展从源头上防治腐败的工作领域。严厉查办在食品药品安全、征地拆迁、企业改制、安全生产、环境保护、重点工程建设、土地出让、社保基金和住房公积金管理等方面的案件，坚决纠正损害群众利益的突出问题。完善制约监督机制，加强对领导干部、人财物管理使用、关键岗位的监督，完善领导干部经济责任审计制度，健全堵塞滋生腐败漏洞的制度体系，有效防止公共权力滥用。自觉加强从政道德修养，严格遵守廉洁从政各项规定。强化监督监察措施，健全发现和惩戒机制，支持纪检、监察机关依法查处腐败案件。

　　(四)深入推进作风转变，切实抓好工作落实。

　　开展学习实践科学发展观活动，牢记“两个务必”，弘扬“八个方面”作风，加强思想作风建设。进一步增强忧患意识，反骄破满，振奋精神，鼓足奋发有为、赶超先进的锐气；进一步增强公仆意识，坚持群众路线，倾听群众呼声，关心群众疾苦，多为群众办实事；进一步增强节俭意识，坚持勤俭办一切事业，努力降低行政成本，建设节约型政府；进一步增强服务意识，倡导扎实深入的工作作风，凝神聚力谋发展、求实效、抓落实。坚持深入基层、深入实际，勇于突破影响科学发展和社会民生的难点问题。严格目标责任制，大力提高办事效率。坚持奖优、治庸、罚劣，鼓励干事创业。

　　各位代表，维护省城稳定，发展省会经济，建设美丽泉城，率先建设全面小康社会，是全市人民的殷切期盼，是时代赋予我们的光荣使命。我们要紧密团结在以胡锦涛同志为总书记的党中央周围，高举中国特色社会主义伟大旗帜，以邓小平理论和“三个代表”重要思想为指导，深入贯彻落实科学发展观，加快构建社会主义和谐社会，认真落实省委、省政府对省会工作的要求，在中共济南市委坚强领导下，解放思想，艰苦奋斗，开拓进取，励精图治，努力开创省会现代化建设新局面！
